# Associations Between Absolute Eosinophil and Basophil Counts and Systemic Immune-Inflammatory Indices in Lung Cancer Patients: A Cross-Sectional Observational Study

**DOI:** 10.7759/cureus.117360

**Published:** 2026-10-02

**Authors:** Olivian Savencu, Horia Liscu, Nicolae I Verga

**Affiliations:** 1 Radiation Oncology, Carol Davila University of Medicine and Pharmacy, Coltea Hospital, Bucharest, ROU

**Keywords:** basophils, complete blood count, eosinophils, lung cancer, monocyte-to-lymphocyte ratio, neutrophil-to-lymphocyte ratio, systemic inflammation

## Abstract

Background and aim: Lung cancer is frequently associated with systemic inflammation and immune dysregulation, reflected by complete blood count (CBC)-derived indices, including the neutrophil-to-lymphocyte ratio (NLR), monocyte-to-lymphocyte ratio (MLR), and platelet-to-lymphocyte ratio (PLR). Eosinophils and basophils are minor leukocyte subpopulations with recognized roles in tumor immunity; however, their associations with classical immune-inflammatory indices remain insufficiently characterized in lung cancer. The aim of this exploratory study was to evaluate associations between absolute eosinophil and basophil counts and CBC-derived immune-inflammatory indices in patients with lung cancer.

Materials and methods: We conducted a retrospective cross-sectional observational study in a cohort of 72 patients with primary lung cancer, evaluated before radiotherapy at a tertiary oncology center. A full differential CBC, including absolute eosinophil and basophil counts, was collected, and NLR, MLR, and PLR were calculated. Associations were assessed using Spearman rank correlation coefficients. Multivariable linear regression models with log-transformed dependent variables were constructed, adjusted for active chemotherapy, age, and sex, using HC3 robust standard errors.

Results: Eosinophils demonstrated significant inverse correlations with NLR (ρ = −0.295, false discovery rate (FDR)-adjusted p-value (p FDR) = 0.024) and MLR (ρ = −0.346, p FDR=0.012). Basophils were inversely associated with NLR (ρ = −0.336, p FDR=0.012); the association with PLR did not retain significance after FDR correction (ρ = −0.233, p FDR=0.073). In the primary multivariable regression, eosinophils were associated with log(MLR) (β = −0.152, 95% CI: −0.250 to −0.053, p=0.004); however, this finding was not robust in sensitivity analyses addressing extreme eosinophil values and distributional skewness. Sensitivity analysis excluding patients on active chemotherapy (n=38) confirmed the eosinophil → NLR association (ρ = −0.448, p=0.005).

Conclusions: In lung cancer patients, absolute eosinophil and basophil counts are inversely associated with CBC-derived immune-inflammatory indices. Eosinophils showed an association with MLR in the primary multivariable model, but this finding was not robust across sensitivity analyses. These findings suggest that minor leukocyte subpopulations may contain information about systemic immune-inflammatory status in lung cancer. Prospective studies are needed to clarify the mechanistic and clinical significance of these associations.

## Introduction

Lung cancer is one of the leading causes of cancer-related mortality worldwide and is frequently characterized by a prominent systemic inflammatory profile. Tumor-associated inflammation extends beyond the local microenvironment and is reflected in alterations of peripheral leukocyte composition, accessible through routine complete blood count (CBC) testing [1].

CBC-derived immune-inflammatory indices -- the neutrophil-to-lymphocyte ratio (NLR), monocyte-to-lymphocyte ratio (MLR), and platelet-to-lymphocyte ratio (PLR) -- are widely used as surrogate markers of systemic immune-inflammatory status in oncology. Elevated values of these indices have been associated with unfavorable prognosis across multiple tumor types, including lung cancer [2-4].

Eosinophils are granulocytic leukocytes involved in inflammatory and immune responses, and accumulating evidence suggests that their peripheral blood levels may have prognostic relevance in cancer. In patients with non-small cell lung cancer (NSCLC) receiving immune checkpoint inhibitors, higher peripheral eosinophil counts or eosinophilia have been associated with more favorable treatment response and survival outcomes [5,6].

Basophils constitute a distinct hematopoietic lineage that matures in the bone marrow and circulates in peripheral blood, developmentally separate from tissue-resident mast cells [7]. They participate in the regulation of type 2 immune responses and have been implicated in tumor-associated immune responses [7].

Nevertheless, associations between absolute eosinophil and basophil counts and classical CBC-derived immune-inflammatory indices remain insufficiently characterized in lung cancer. Characterizing whether these minor leukocyte subpopulations covary with lymphocytes and established CBC-derived indices may provide a more detailed description of systemic inflammatory patterns in lung cancer using routinely available CBC data.

From a systems medicine perspective, peripheral blood can be conceptualized as an integrative subsystem in which inflammatory, immune, and metabolic components converge and interact [8]. Previous studies have demonstrated associations between CBC-derived immune-inflammatory indices and other physiological domains in patients with lung cancer [9].

The primary objective of this exploratory study was to evaluate the cross-sectional associations between absolute eosinophil and basophil counts and three CBC-derived immune-inflammatory indices (NLR, MLR, and PLR) in adults with primary lung cancer evaluated before radiotherapy. The six corresponding correlations constituted the primary analytical family, with no single exposure-outcome association designated as primary. Additional exploratory and sensitivity analyses were performed to further characterize these relationships and assess their robustness.

## Materials and methods

Study design and population

We conducted a retrospective cross-sectional observational study, including adult patients with a confirmed diagnosis of primary lung cancer, evaluated at MedEuropa Oradea, a tertiary oncology center in Oradea, Romania. Data were retrieved from electronic medical records as part of routine clinical care. The study was approved by the Institutional Ethics Committee (Decision Med039/22.01.2026) and conducted in accordance with the principles of the Declaration of Helsinki. All data were anonymized before analysis. The study included patients evaluated between 2016 and 2025. Patients were identified by searching the institutional database for records of patients with a diagnosis of lung cancer. Sampling was non-consecutive. No formal a priori sample size calculation was performed.

Inclusion and exclusion criteria

Patients were eligible for inclusion if they met all of the following criteria: (1) confirmed diagnosis of primary lung cancer (squamous cell carcinoma, adenocarcinoma, pulmonary neuroendocrine tumors -- including small cell lung cancer and other neuroendocrine carcinomas -- or histologically unspecified NSCLC); (2) age ≥18 years; (3) complete differential CBC available, including absolute eosinophil and basophil counts; and (4) blood samples obtained before initiation of radiotherapy. 

Patients were excluded if they had pulmonary metastases from other non-pulmonary primary tumors, documented blood sampling during or after initiation of radiotherapy, or incomplete laboratory data for the variables of interest. When multiple pre-radiotherapy CBC measurements were available for a given patient, the laboratory profile obtained closest to the initiation of radiotherapy was selected for analysis. For five patients, the available retrospective records did not permit confirmation of CBC timing relative to radiotherapy initiation.

Laboratory variables

Laboratory data were obtained from blood tests performed as part of routine clinical care. For each patient, the CBC obtained closest to the initiation of radiotherapy was selected for statistical analysis. For patients with documented sampling dates, the selected CBC was obtained 1-3 days before radiotherapy initiation, in accordance with the institutional pre-radiotherapy assessment workflow. All hematological parameters and derived immune-inflammatory indices used in the statistical analyses were obtained or calculated from the same laboratory profile. Analyzed variables included absolute counts of neutrophils, lymphocytes, monocytes, platelets, eosinophils, and basophils (cells/µL). Immune-inflammatory indices were calculated as: $\small \mathrm{NLR}=\mathrm{Neutrophils}/\mathrm{Lymphocytes}$; $\small \mathrm{MLR}=\mathrm{Monocytes}/\mathrm{Lymphocytes}$; $\small \mathrm{PLR}=\mathrm{Platelets}/\mathrm{Lymphocytes}$. Active chemotherapy at the time of blood sampling (CT) was documented as a binary variable (0/1). Active chemotherapy was defined as documentation in the medical record indicating that the patient was currently receiving a chemotherapy regimen, such as documentation of an ongoing treatment cycle. The exact interval between the most recent chemotherapy administration and CBC sampling was not consistently available in the extracted dataset.

Statistical analysis

All analyses were performed in Python 3.13.5 using pandas 2.2.3, NumPy 2.3.5, SciPy 1.17.0, and statsmodels 0.14.6. The primary analyses comprised six Spearman correlations evaluating the associations of absolute eosinophil and basophil counts with NLR, MLR, and PLR. These six correlations constituted a single primary analytical family, with no individual exposure-outcome association designated as primary. This analytical hierarchy was not prospectively prespecified. False discovery rate (FDR) correction using the Benjamini-Hochberg procedure was applied across these six correlations to account for multiple testing, and FDR-adjusted p-values (p FDR) are reported alongside unadjusted p-values. Correlations involving lymphocyte count and partial correlations controlling for lymphocyte count were considered exploratory and interpreted without formal FDR correction. Partial Spearman correlations controlling for lymphocyte count were calculated by rank-transforming the variables, residualizing the ranked variables of interest with respect to ranked lymphocyte count, and calculating the correlation between the resulting residuals. The six eosinophil-to-lymphocyte ratio (ELR) and basophil-to-lymphocyte ratio (BLR) correlations constituted a separate exploratory family and were FDR-corrected within that family. Differences in NLR, MLR, and PLR across eosinophil and basophil tertiles were evaluated using the Kruskal-Wallis test. Tertile-based analyses and multivariable regression models were considered exploratory analyses, while analyses examining the influence of chemotherapy status, extreme values, and variable transformations were considered sensitivity analyses. A two-sided p-value <0.05 was considered statistically significant. Variables included in the multivariable models were selected based on clinical relevance rather than according to a univariate p-value threshold. Eosinophil and basophil counts were included as the variables of interest, while age, sex, and active chemotherapy at the time of blood sampling were included as potential confounding variables. NLR, MLR, and PLR were natural-log transformed before multivariable regression. Continuous predictors (absolute eosinophil count, absolute basophil count, and age) were standardized, whereas sex and active chemotherapy were entered as binary indicator variables. Multivariable analyses were performed using complete cases for the included covariates; because chemotherapy status was undocumented in five patients, these models included 67 patients. HC3 heteroscedasticity-robust standard errors were used. Multicollinearity was assessed using variance inflation factors (VIFs), and potentially influential observations were evaluated using Cook's distance. Because eosinophil counts included zero values, the sensitivity analysis involving transformation of eosinophil counts used ln(1 + eosinophil count).

## Results

Cohort characteristics

Of 112 initially screened patients, 72 met the inclusion criteria and constituted the final analytic cohort (20 excluded for non-pulmonary cancer, 5 for documented non-pre-radiotherapy timing, and 15 for incomplete data). For five additional patients, sampling timing could not be confirmed from available medical records; these were retained in the analysis given that pre-radiotherapy assessment represented the standard institutional workflow. The baseline demographic and clinical characteristics of the study population are summarized in Table 1.

**Table 1:** Baseline characteristics of the study cohort (n=72) ^a^NSCLC NOS includes patients with a confirmed bronchopulmonary malignancy (non-SCLC) in whom the precise histological subtype could not be determined from available medical records. Percentages may not total exactly 100% due to rounding. CT, active chemotherapy at time of blood sampling; IQR, interquartile range; NSCLC NOS, non-small cell lung cancer, not otherwise specified; SCLC, small cell lung cancer.

Characteristic	Value
Age, years, median (IQR)	65.0 (60.0-70.0)
Male sex, n (%)	56 (77.8%)
Female sex, n (%)	16 (22.2%)
Cancer type, n (%)	
Squamous cell carcinoma	29 (40.3%)
Adenocarcinoma	20 (27.8%)
Neuroendocrine tumors (total)	19 (26.4%)
Small cell lung cancer	15 (20.8%)
Other neuroendocrine tumors	4 (5.6%)
NSCLC NOS^a^	4 (5.6%)
Active chemotherapy (CT=1), n (%)	29 (40.3%)
No chemotherapy (CT=0), n (%)	38 (52.8%)
CT undocumented, n	5 (6.9%)
Pre-radiotherapy timing documented, n (%)	67 (93.1%)
Timing undocumented, n	5 (6.9%)

Laboratory variable profiles

All continuous variables demonstrated non-normal distributions (Shapiro-Wilk test, p<0.05 for all). Descriptive statistics for hematological parameters are presented in Table 2.

**Table 2:** Hematological parameters of the cohort (n=72) IQR, interquartile range; MLR, monocyte-to-lymphocyte ratio; NLR, neutrophil-to-lymphocyte ratio; PLR, platelet-to-lymphocyte ratio; SD, standard deviation.

Parameter	Mean ± SD	Median (IQR)	Min-max
Eosinophils (/µL)	188.9 ± 461.4	90 (50-150)	0-3,100
Basophils (/µL)	39.5 ± 34.0	30 (20-59)	0-200
Neutrophils (/µL)	6,004 ± 2,851	5,585 (3,630-7,273)	2,170-14,400
Lymphocytes (/µL)	1,634 ± 718	1,530 (1,130-2,160)	260-4,360
Monocytes (/µL)	770 ± 462	660 (500-895)	61-2,730
Platelets (/µL)	300,485 ± 118,993	270,500 (222,000-358,000)	76,700-606,000
NLR	4.33 ± 2.62	3.45 (2.67-5.31)	0.90-11.58
MLR	0.55 ± 0.40	0.45 (0.31-0.67)	0.06-2.26
PLR	224.0 ± 172.7	186.9 (122.8-259.6)	55.8-1357.7

Univariate associations

Eosinophils demonstrated significant inverse correlations with NLR (ρ = −0.295, p=0.012) and MLR (ρ = −0.346, p=0.003), without a significant association with PLR (ρ = −0.124, p=0.299). Basophils were inversely associated with NLR (ρ = −0.336, p=0.004) and PLR (ρ = −0.233, p=0.049), without a significant association with MLR (ρ = −0.083, p=0.486). Spearman correlation coefficients are presented in Table 3. Scatterplots illustrating the associations between eosinophil count and MLR and between basophil count and NLR are presented in Figure 1.

**Table 3:** Spearman correlations between eosinophils/basophils and immune-inflammatory indices (n=72) *p FDR<0.05. ρ, Spearman rank correlation coefficient; MLR, monocyte-to-lymphocyte ratio; NLR, neutrophil-to-lymphocyte ratio; p FDR, false discovery rate-adjusted p-value (Benjamini-Hochberg procedure); PLR, platelet-to-lymphocyte ratio.

Association	Spearman ρ	p-value	p FDR	N
Eosinophils → NLR	−0.295	0.012	0.024*	72
Eosinophils → MLR	−0.346	0.003	0.012*	72
Eosinophils → PLR	−0.124	0.299	0.358	72
Basophils → NLR	−0.336	0.004	0.012*	72
Basophils → MLR	−0.083	0.486	0.486	72
Basophils → PLR	−0.233	0.049	0.073	72

**Figure 1:**
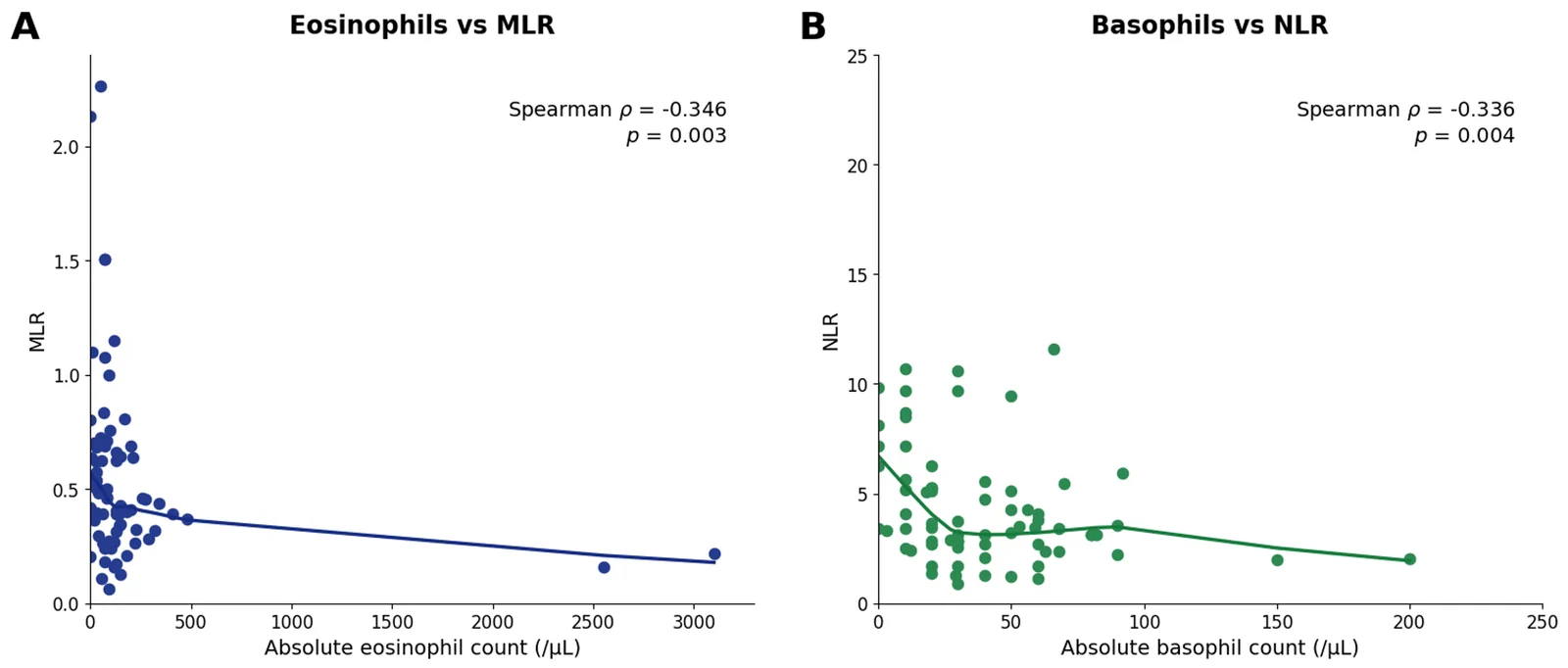
Scatterplots illustrating the associations between (A) absolute eosinophil count and monocyte-to-lymphocyte ratio (MLR) and (B) absolute basophil count and neutrophil-to-lymphocyte ratio (NLR) in patients with lung cancer Each point represents an individual patient. Solid lines represent locally weighted smoothing (LOESS). Spearman correlation coefficients (ρ) and corresponding p-values are shown within each panel.

Tertile analysis of basophil counts (T1≤20/µL, T2=20-50/µL, and T3>50/µL) revealed significant between-group differences in NLR (T1: 5.13, T2: 2.88, and T3: 3.13; Kruskal-Wallis p=0.014). Given the non-monotonic pattern across tertiles, this finding is more consistent with a possible threshold pattern at low basophil counts than with a graded dose-response relationship. Eosinophil tertiles did not demonstrate a significant gradient for any index (p>0.05 for all).

To further characterize the structural relationships within the leukocyte differential, we examined correlations between eosinophil and basophil counts and absolute lymphocyte count. Both eosinophils (ρ = +0.305, p=0.009) and basophils (ρ = +0.264, p=0.025) covaried positively with lymphocyte count. Lymphocyte count was itself strongly and inversely associated with all three immune-inflammatory indices (NLR: ρ = −0.618; MLR: ρ = −0.640; PLR: ρ = −0.715; all p<0.001). Partial correlation analysis, controlling for lymphocyte count, substantially attenuated the eosinophil → MLR association (partial ρ = −0.143, p=0.231; 59% attenuation) and partially attenuated the basophil → NLR association (partial ρ = −0.233, p=0.047; 31% attenuation), suggesting that lymphocyte dynamics contribute to, but do not fully account for, the observed inverse associations (Table 4).

**Table 4:** Spearman correlations between eosinophil/basophil counts and lymphocyte count, and partial correlation analysis (n=72) *p<0.05; **p<0.01; ***p<0.001. Partial correlations were computed as Spearman correlations of residuals after linear regression on lymphocyte count. ρ, Spearman rank correlation coefficient; NLR, neutrophil-to-lymphocyte ratio; MLR, monocyte-to-lymphocyte ratio; PLR, platelet-to-lymphocyte ratio.

Association	Spearman ρ	p-value		N
Eosinophils and basophils vs. lymphocyte count				
Eosinophils → lymphocytes	+0.305	0.009**	72	
Basophils → lymphocytes	+0.264	0.025*	72	
Lymphocyte count vs. immune-inflammatory indices				
Lymphocytes → NLR	−0.618	<0.001***	72	
Lymphocytes → MLR	−0.640	<0.001***	72	
Lymphocytes → PLR	−0.715	<0.001***	72	
Partial correlations controlling for lymphocyte count				
Eosinophils → MLR (simple)	−0.346	0.003**	72	
Eosinophils → MLR (partial)	−0.143	0.231	72	
Basophils → NLR (simple)	−0.336	0.004**	72	
Basophils → NLR (partial)	−0.233	0.047*	72	

To further explore whether lymphocyte normalization affects the observed relationships, we computed lymphocyte-normalized ratios $\small \mathrm{ELR}=\mathrm{Eosinophils}/\mathrm{Lymphocytes}$; $\small \mathrm{BLR}=\mathrm{Basophils}/\mathrm{Lymphocytes}$ and examined their associations with immune-inflammatory indices. Median ELR was 0.058 (interquartile range (IQR): 0.032-0.111) and median BLR was 0.021 (IQR: 0.010-0.033). Neither ELR nor BLR demonstrated significant associations with NLR, MLR, or PLR after FDR correction within this exploratory family of six comparisons (Table 5). These analyses are presented as complementary exploratory analyses and do not redefine the six primary absolute-count correlations reported in Table 3.

**Table 5:** Exploratory Spearman correlations between lymphocyte-normalized ratios (ELR and BLR) and immune-inflammatory indices (n=72) ρ, Spearman rank correlation coefficient; BLR, basophil-to-lymphocyte ratio; ELR, eosinophil-to-lymphocyte ratio; MLR, monocyte-to-lymphocyte ratio; NLR, neutrophil-to-lymphocyte ratio; p FDR, false discovery rate-adjusted p-value (Benjamini-Hochberg procedure applied across the six exploratory ELR/BLR comparisons); PLR, platelet-to-lymphocyte ratio.

Association	Spearman ρ	p-value	p FDR	N
ELR → NLR	−0.039	0.748	0.748	72
ELR → MLR	−0.058	0.631	0.748	72
ELR → PLR	+0.172	0.149	0.447	72
BLR → NLR	−0.090	0.453	0.748	72
BLR → MLR	+0.187	0.115	0.447	72
BLR → PLR	+0.055	0.645	0.748	72

Multivariable regression

In the multivariable regression model for log(MLR), eosinophils remained independently associated (β = −0.152, 95% CI: −0.250 to −0.053, p=0.004), after adjustment for active chemotherapy, age, and sex (Table 6). VIFs ranged from 1.12 to 1.61, indicating no evidence of problematic multicollinearity. Examination of Cook's distance identified potentially influential observations, particularly in the NLR model; therefore, the results were interpreted together with the sensitivity analyses addressing extreme eosinophil values. Active chemotherapy contributed significantly to MLR variability (β = +0.553, p=0.002), confirming its relevance as an adjustment variable. Basophils did not demonstrate a significant independent association in any model. Models for log(NLR) and log(PLR) identified no significant independent predictors among the variables of interest.

**Table 6:** Multivariable linear regression -- outcome: log(MLR), n=67 **p<0.01. HC3 robust standard errors. β, standardized regression coefficient; MLR, monocyte-to-lymphocyte ratio.

Variable	β	95% CI	p-value	
Outcome: log(MLR), n=67, R²=0.247				
Eosinophils (std)	−0.152	−0.250 to −0.053	0.004**	
Basophils (std)	+0.058	−0.160 to +0.276	0.606	
Chemotherapy (CT)	+0.553	+0.224 to +0.882	0.002**	
Age (std)	+0.084	−0.098 to +0.266	0.368	
Sex (male)	−0.077	−0.343 to +0.189	0.571	

Sensitivity analysis

In patients without documented active chemotherapy (CT=0, n=38), an inverse eosinophil-NLR association (ρ = −0.448, p=0.005) and an inverse basophil-NLR association (ρ = −0.325, p = 0.047) were observed. Associations with MLR and PLR did not reach statistical significance in this subgroup. These exploratory subgroup analyses were not adjusted for multiple comparisons and should therefore be interpreted cautiously.

Because the absolute eosinophil distribution was markedly right-skewed and contained two extreme values (2,550 and 3,100 /µL; median: 90 /µL), additional sensitivity analyses were performed. The primary rank-based associations were retained after exclusion of these observations (eosinophil-MLR: ρ = −0.302, p=0.011; eosinophil-NLR: ρ = −0.248, p=0.038; n=70). In contrast, the eosinophil coefficient in the multivariable model was sensitive to the handling of the eosinophil distribution: after exclusion of the two extreme values, it was no longer significant (β = −0.056, p=0.363), and it was also non-significant when eosinophil counts were log-transformed (β = −0.103, p=0.307). Thus, the multivariable eosinophil-MLR finding should be considered model-sensitive rather than a robust independent effect.

## Discussion

Principal findings

In this cohort of lung cancer patients evaluated before radiotherapy, absolute eosinophil and basophil counts demonstrated weak inverse associations with selected CBC-derived immune-inflammatory indices. Eosinophils were inversely associated with NLR and MLR after FDR correction, with the MLR association also reaching significance in the exploratory multivariable model; however, this multivariable finding was not robust in sensitivity analyses addressing the markedly right-skewed eosinophil distribution and extreme observations. Basophils were inversely associated with NLR after FDR correction, whereas the association with PLR did not remain significant after FDR correction; significant between-tertile differences were observed for NLR. Inverse NLR associations were also observed in the exploratory subgroup analysis restricted to patients without documented active chemotherapy, although these subgroup analyses were not adjusted for multiple comparisons and should be interpreted cautiously.

Eosinophils in the context of systemic inflammation

The inverse association between eosinophils and MLR was significant in the exploratory multivariable model, although this finding was sensitive to alternative handling of the markedly right-skewed eosinophil distribution and should therefore be interpreted cautiously. Peripheral eosinophils are frequently reduced in severe systemic inflammatory states, and eosinopenia has been associated with unfavorable outcomes in inflammatory clinical settings [10]. In lung cancer, prior studies have reported that higher peripheral eosinophil counts or eosinophilia are associated with more favorable responses to immunotherapy and longer survival [5,6]. These observations provide clinical context for further investigation of the relationship between eosinophil counts and systemic immune-inflammatory indices.

Recent studies have evaluated eosinophils together with conventional inflammatory indices in NSCLC. In patients receiving neoadjuvant immunotherapy, post-treatment eosinophil fractions were higher and NLR, PLR, and MLR values were lower among patients achieving a major pathological response, whereas pretreatment eosinophil differences were less evident [11]. Other data indicate that baseline absolute eosinophil counts may provide prognostic information alongside conventional inflammatory indices such as NLR, LMR, and PLR [12]. Although these studies differ from the present pre-radiotherapy cross-sectional analysis, they support further investigation of the relationship between eosinophils and composite systemic inflammatory indices.

Analysis of the internal structure of the leukocyte differential in the present data suggests that eosinophils covary positively with lymphocytes rather than with myeloid cells. This suggests that the inverse eosinophil-MLR association may partly reflect shared variation with lymphocyte count: a tumor-related inflammatory environment associated with lymphopenia may also be associated with low eosinophil counts and elevated MLR. Interpreting these associations as direct relationships between subpopulations would therefore be incorrect; they more likely reflect covariation of these cells within a perturbed systemic immune context.

Basophils and systemic immune-inflammatory status

The basophil-NLR association, which retained significance after FDR correction (ρ = −0.336, p FDR=0.012) and was accompanied by significant between-tertile differences in NLR, suggests that patients with lower basophil counts tend to have higher NLR. Basophils are type 2 immune cells, and lower counts in the context of elevated NLR may reflect alterations in systemic immune-inflammatory balance. The association between basophils and PLR did not retain significance after FDR correction (p FDR=0.073), and the basophil-NLR association did not withstand multivariable adjustment, indicating that the observed effects may be influenced by other factors, including active chemotherapy and lymphocyte dynamics [7,13].

A previous study in patients with bronchial carcinoma reported that peripheral basophils were significantly elevated in tumor-bearing patients compared with healthy individuals, with the highest values in squamous cell carcinoma [14]. Differences from the present findings may reflect differences in disease stage, histological subtype, and treatment status.

Evidence regarding circulating basophils in lung cancer remains limited. In a recent retrospective surgical cohort, perioperative changes in peripheral blood basophil percentage were independently associated with overall survival, with postoperative decreases associated with more favorable outcomes [15]. Although that study evaluated longitudinal perioperative changes rather than cross-sectional associations with inflammatory indices, it supports further investigation of circulating basophils in lung cancer.

Specificity of absolute counts versus ratios

A methodologically relevant observation is that derived ratios ELR (eosinophils/lymphocytes; median: 0.058, IQR: 0.032-0.111) and BLR (basophils/lymphocytes; median: 0.021, IQR: 0.010-0.033) did not demonstrate significant associations with NLR, MLR, or PLR after FDR correction (all p FDR>0.14; Table 5). This suggests that, within the present dataset, the observed associations were more apparent when considering absolute eosinophil and basophil counts rather than lymphocyte-normalized ratios.

When normalizing for lymphocytes, the signal disappears, which is consistent with an important contribution of lymphocyte-related covariance to the observed associations rather than a direct relationship between the cell populations. Because NLR, MLR, and PLR share lymphocyte count as their denominator and all investigated cell counts were obtained from the same CBC, the observed associations may partly reflect mathematical coupling and shared variation within the leukocyte differential rather than independent biological relationships.

Limitations

The retrospective cross-sectional design precludes causal inference and temporal interpretation. The study did not evaluate treatment response, survival outcomes, or the incremental clinical utility of eosinophil or basophil counts beyond conventional CBC-derived indices. The cohort size is modest (n = 72), with a sensitivity analysis limited to n = 38, reducing statistical power for detecting small effects. Systemic corticosteroid use, an important confounder for eosinophil and basophil counts, was not systematically documented and could not be controlled. Detailed information on systemic treatment regimens and the exact interval between the most recent chemotherapy administration and CBC sampling was not consistently available in the retrospective records, limiting assessment of treatment-related effects on the investigated hematological parameters. Similarly, comorbidities independently influencing eosinophils (allergies, parasitic diseases, autoimmune conditions, and COPD) were not available from retrospective records. The histological heterogeneity of the cohort, including NSCLC subtypes, SCLC, and neuroendocrine tumors, may introduce additional biological variability. These limitations require that findings be interpreted as exploratory and hypothesis-generating for prospective studies. The absolute eosinophil distribution was markedly right-skewed and contained two extreme values; although the primary rank-based correlations were robust to their exclusion, the multivariable eosinophil estimate was sensitive to outlier exclusion and transformation, limiting inference regarding an independent effect.

## Conclusions

In lung cancer patients evaluated before radiotherapy, absolute eosinophil and basophil counts showed weak inverse associations with selected CBC-derived immune-inflammatory indices. The eosinophil-MLR association was statistically significant in the exploratory multivariable model, although this finding was not robust to sensitivity analyses addressing extreme eosinophil values and distributional skewness. The observed associations may partly reflect shared lymphocyte-related covariance and mathematical coupling among variables derived from the same CBC rather than independent biological relationships. Mechanistic interpretation is limited by the study design, and the findings do not establish directionality, causality, prognostic value, or incremental clinical utility. Given the retrospective cross-sectional design and modest sample size, these findings should be considered exploratory and hypothesis-generating. Prospective studies incorporating data on corticosteroid use, detailed immune profiling, and treatment characteristics are needed to clarify the clinical and mechanistic implications of these observations.
